# A Systematic Review and Meta-analysis of Timing and Outcome of Intestinal Failure Surgery in Patients with Enteric Fistula

**DOI:** 10.1007/s00268-017-4224-z

**Published:** 2017-09-18

**Authors:** Fleur E. E. de Vries, Jasper J. Atema, Oddeke van Ruler, Carolynne J. Vaizey, Mireille J. Serlie, Marja A. Boermeester

**Affiliations:** 1 Department of Surgery, Academic Medical Centre Amsterdam, PO Box 22660, Meibergdreef 9, 1105 AZ Amsterdam, The Netherlands; 2 Department of Surgery, Onze Lieve Vrouwe Gasthuis, Jan Tooropstraat 164, 1061 AE Amsterdam, The Netherlands; 3 Department of Surgery, IJsselland Hospital, Prins Constantijnweg 2, 2906 ZC Capelle aan den IJssel, The Netherlands; 4 Department of Surgery, St Marks Hospital, Watford Road, Harrow, London, HA1 3UJ UK; 5 Department of Endocrinology and Metabolism, Academic Medical Centre Amsterdam, Meibergdreef 9, 1105 AZ Amsterdam, The Netherlands

## Abstract

**Background:**

The timing of intestinal failure (IF) surgery has changed. Most specialized centers now recommend postponing reconstructive surgery for enteric fistula and emphasize that abdominal sepsis has to be resolved and the patient’s condition improved. Our aim was to study the outcome of postponed surgery, to identify risk factors for recurrence and mortality, and to define more precisely the optimal timing of reconstructive surgery.

**Methods:**

PubMed, Embase, and the Cochrane Library were systematically reviewed on the outcomes of reconstructive IF surgery (fistula recurrence, mortality, morbidity, hernia recurrence, total closure, enteral autonomy). If appropriate, meta-analyses were performed. Optimal timing was explored, and risk factors for recurrence and mortality were identified.

**Results:**

Fifteen studies were included. The weighted pooled fistula recurrence rate was 19% (95% CI 15–24). Lower recurrence rates were found in studies with a longer median time and/or, at the minimum of the range, a longer time interval to surgery. Overall mortality was 3% (95% CI 2–5). Total fistula closure rates ranged from 80 to 97%. Enteral autonomy after reconstructive surgery, mentioned in four studies, varied between 79 and 100%.

**Conclusions:**

Postponed IF surgery for enteric fistula is associated with lower recurrence. Due to the wide range of time to definitive surgery within each study, optimal timing of surgery could not be defined from published data.

## Introduction

An enterocutaneous fistula (ECF) is an unnatural communication between the gastrointestinal tract and the skin. Enteroatmospheric fistulas (EAF), with visible mucosa and the absence of overlying soft-tissue within an open abdomen, form a special subset of ECFs. Approximately 75–85% of such fistulas arise as complications following abdominal surgery [1]. Although ECF and EAF are rare, they pose complex and challenging problems. The most important homeostatic and metabolic challenge arising from ECF or EAF is intestinal failure (IF). ECF/AEF patients usually have type 2 IF [2]. Due to massive fluid and electrolyte losses and reduced nutrient resorption, these patients frequently rely on parenteral nutrition (PN) to fulfill their nutritional demands. Sepsis elimination, fistula output reduction, wound care, homeostasis and adequate nutritional support are the cornerstones of treatment during the so-called bridging-to-surgery period for which recommendations are given in the IF Guidelines of the European Society of Coloproctology [2].

The first reports on delaying reconstructive surgery for ECF/EAF appeared in the 1970s. It was not until 1983, however, that a step-by-step strategy of ECF management involving postponed ECF surgery was described [3, 4]. It was recommended that surgery should be delayed by as many as 6–8 weeks until all signs of sepsis had disappeared and the patient had been restored to nutritional health. However, it took another 20 years for the first reports to be published on the management of patients according to this strategy.

A reason to delay surgery in the case of ECF is the chance of spontaneous closure, which is most likely to occur in low-output fistula and within 3–6 months; EAF never close spontaneously. Postponed reconstructive surgery also allows ample time for patient recovery, fistula maturation, resolution of abdominal inflammation, and softening of adhesions and scar tissue formation on an open abdomen, enabling safe adhesiolysis. Nowadays, this is common practice in dedicated IF centers but not embraced in general.

The aim of this review was threefold. Our primary goal was to systematically review the available literature on fistula recurrence rates and secondary outcomes of postponed reconstructive surgery for ECF/EAF. As ECF/EAF patient care has improved over the years, our review excluded studies that compared outcomes with historical cohorts. Our second goal was to define the optimal timing of reconstructive surgery for patients with ECF/EAF. Finally, we aimed to identify risk factors for fistula recurrence and mortality.

## Materials and methods

The PRISMA (Preferred Reporting Items for Systematic Reviews and Meta-Analyses) guidelines [5] and the MOOSE (Meta-Analysis of Observational Studies in Epidemiology) checklist [6] were followed.

### Definitions

Postponed reconstructive surgery was defined as single-staged, elective surgery for ECF/EAF takedown in intestinal fistula patients which was delayed by a certain period until sepsis had resolved and the patient had regained the best achievable physical condition. Short-term mortality was defined as either 30-day mortality or in-hospital mortality. We did not limit reporting on morbidity to one specific classification system. Total fistula closure rate included successful primary ECF/EAF takedowns, closures after repetitive surgery after recurrence, and spontaneous closures after recurrence. Hernia recurrence was defined as a ventral hernia after abdominal wall reconstruction. Enteral autonomy was defined as the successful discontinuation of all types of artificial nutritional support including parenteral nutrition and intravenous fluids and electrolytes, following the definition of the Association of Surgeons of Great Britain and Ireland (ASGBI) [7].

### Search

On August 24, 2016, a systematic search was performed for articles on the outcomes of elective surgery in patients with enterocutaneous fistula and/or enteroatmospheric fistula. The primary outcome was fistula recurrence, while secondary outcomes were mortality, morbidity, total closure rate, hernia recurrence, and enteral autonomy. We involved a clinical librarian to optimize the search strategy. We used MEDLINE (PubMed), Embase (Ovid), and the Cochrane Library to identify related studies. Our search terms included enterocutaneous fistula, enteroatmospheric fistula, intestinal failure, surgical treatment, closure, recurrence, mortality, morbidity, and complications. Both MESH terms and free text were used. “Appendix 1” contains the complete search. The search was not limited to year of publication or language. The authors F.V. and J.A. independently screened all titles and abstracts. References of the included studies were cross-checked for other relevant studies.

### Inclusion and exclusion criteria

Studies reporting on 25 patients or more, and that addressed elective ECF/EAF takedowns as per the definition, were included. The principles of elective surgery had to be provided in the methods section. Additionally, studies at least had to report on present review’s primary outcome, i.e., fistula recurrence rate. Only studies on small bowel and/or colon fistulas were included. Studies on pediatric patients and studies addressing biliary, pancreatic, or anal fistula surgery were excluded.

### Quality assessment

The modified Methodological Index for Non-Randomized Studies (MINORS) [8] was used to assess the methodological quality of all studies (“Appendix 2”). A maximum of 14 points could be achieved.

### Data extraction

Data on primary and, whenever possible, secondary outcomes were extracted from the text. Study characteristics, i.e., year of publication, type of study, inclusion period, number of elective patients, median time to surgery, and follow-up were also retrieved from the text. Significant risk factors for recurrence and mortality were identified in the individual studies.

### Statistical analysis

SPSS statistics, version 21.0, was used. Descriptive analyses were used to review the identified studies, and if appropriate, meta-analyses were conducted using RStudio statistics, version 2.13.1, and studies were pooled within a random effects model.

## Results

**Fig. 1:**
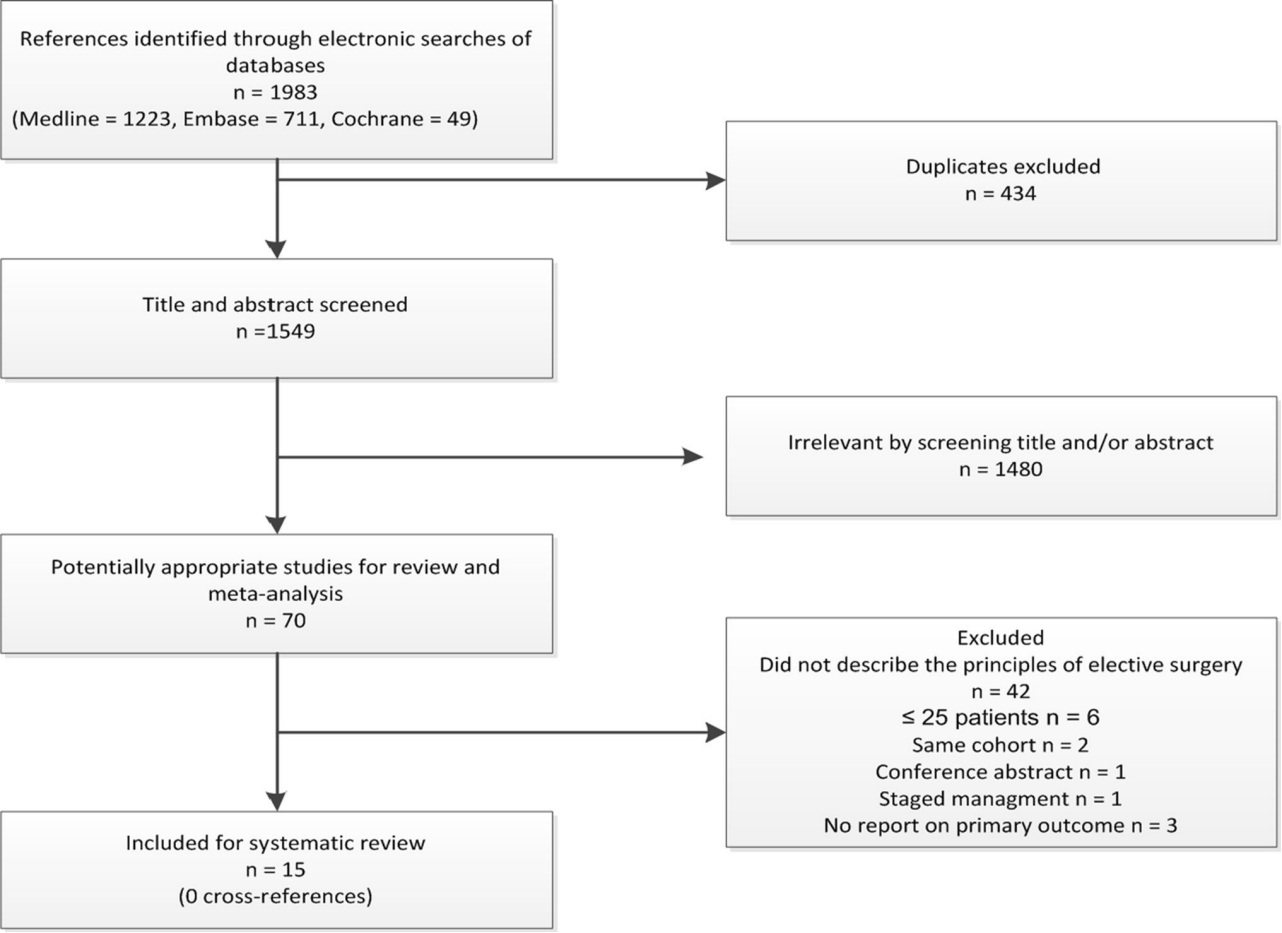
Flowchart of the systematic review

Out of 1549 articles initially identified by the search, 70 were selected for a full text review (Fig. 1). Fifty-five articles were excluded for the following reasons: forty-two articles did not describe the criteria for elective surgery nor did they report separately on the results of acute and elective surgery. Six studies included less than 25 elective patients [9–14]. Four studies reported on two identical or overlapping cohorts [15–18] and were, therefore, combined for the purpose of our analysis. One study described staged management [19], and three other studies did not report on this review’s primary outcome [20–22]. One study was a conference abstract. Finally, fifteen studies (10 retrospective cohort studies [16, 17, 23–30] and five prospective cohort studies [31–35]) were included, comprising a total of 1380 patients who had undergone elective ECF/EAF surgery. Included studies were published between 2004 and 2016. Table 1 presents the characteristics and outcomes of the included studies.

**Table 1:** Study characteristics and outcome

Study	Type of cohort	Modified MINORS score	Inclusion period	Elective patients	Patients with TPN (%)	Median time to surgery in days (range)	Follow-up	ECF recurrence	Short-term mortality	Morbidity	Hernia recurrence	Final fistula closure	Enteral autonomy
Martinez [34]	Prospective	9	2005–2009	71	93%	63 (5–979)	N/A	31%^b^	NA (20% overall^b^)	N/A	N/A	94%	N/A
Visschers [15, 16]	Retrospective	9	1990–2010	148	59%	72 (4–270)	N/A	16%^b^	7%	76% overall	N/A	96%	N/A
Martinez [35]	Prospective	9	2011–2013	50	74%	115 (2–1120)	N/A	38%^b^	0%	N/A	N/A	80%	N/A
Wainstain [30]	Retrospective	6	2002–2014	47	100%	175 (35–469)	N/A	15%^b^	4%	75% postoperative complications	N/A	91%	N/A
Brenner [24]	Retrospective	9	1989–2005	135	69%	175 (7–1456)	N/A	17%^b^	3%	N/A	N/A	84%	N/A
Lynch [23]	Retrospective	9	1994–2001	203	36%	180 (0–840)	Median 9.5 months	21%^a^	3%^a^	6% reoperations	N/A	N/A	N/A
Hollington [18]/Mawdsley [17]	Retrospective	12	1992–2002	167	52% in complete cohort including conservative managed patients	240 (30–5400)	Median 18 months (1–137)	33%^b^	4%	N/A	N/A	82%	N/A
Ravindran [27]	Retrospective	10	2000–2010	41	52%	250 (98–810)	N/A	5%^a^	0%	86% overall	N/A	95%	95%
Owen [25]	Retrospective	10	1987–2010	153	80%	267 (0–2201)	N/A	29%^d^	4%	88% overall 31% SSI	N/A	84%	86%
Datta [32]	Prospective	14	2005–2007	35	35%	270 (180–440)	6-months	11%^c^	3%	N/A	N/A	89%	100%
Atema [29]	Retrospective	12	2011–2014	44	100%	270 (90–780)	Median 8 months (1–43)	16% (7% incl 2nd attempt)	5%	36% Clavien–Dindo Grade III or IV	N/A	93%	79%
Connoly [31]	Prospective	13	1999–2006	61	N/A	330 (180–1020)	16–84 months	11%^b^	5%	83% overall 38% SSI 3% reoperation	29%	N/A	N/A
Rahbour [26]	Retrospective	9	2003–2009	149	33%	360 (30–5100)	Median 22.8 months	14%^d^	0%	N/A	N/A	95%	N/A
Slater [28]	Retrospective	11	2000–2009	39	N/A	> 3 months	3 years	5%^d^	3%	72% overall 21% SSI	36%	N/A	N/A
Krpata [33]	Prospective	11	2005–2012	37	N/A	Elective	Mean 20 months (3–73)	14%^d^	3%	65% SSI	32%	97%	N/A
Total				1380									

^a^3-Month follow-up

^b^Any point of time during follow-up

^c^6-Month follow-up

^d^30-Day follow-up

*ECF* enterocutaneous fistula, *EAF* enteroatmospheric fistula, *SSI* surgical site infection, *TPN* total parenteral nutrition

### Baseline characteristics of included studies

The mean or median age varied between 48 [26] and 61 [28] years. Studies were comparable for the included percentage of small bowel fistulas, being more than 65% in each study. Other fistulas included were colonic fistulas, and four studies [24–26, 34] included gastric fistulas (less than 6% of the fistulas). The etiologies of the fistulas were comparable in most studies, with more than 75% of the patients having fistulas as a result of complicated abdominal surgery. Only one study showed a lower percentage (50%) of postoperative fistulas [27]. The percentage of patients with inflammatory bowel disease (IBD) varied between 10% [25] and 50% [23]. Most studies included both simple and complex fistulas such as EAF, and low as well as high-output fistulas. Wainstein [30] focused on patients with EAF only, and the study of Martinez [34] included 84% EAF. Connoly et al. [31] included fistulas within the open abdomen. Two other studies [28, 33] focused on ECF takedown and simultaneous large complex hernia repair. It is likely that these studies included more complex fistulas than the other studies, but this was difficult to determine. Not all studies reported on all outcome parameters, and therefore, the number of studies included in each meta-analysis varied.

### Quality of the studies included

All the studies included were scored using MINORS, and the scores ranged from 6 to 14 points (maximum possible score is 14, Table 1). No studies were excluded after scoring.

### Outcomes

#### ECF recurrence

**Fig. 2:**
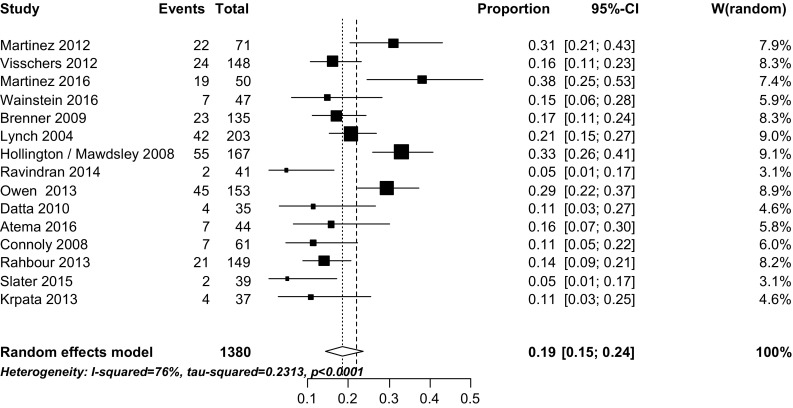
Weighted pooled ECF recurrence rates

**Fig. 3:**
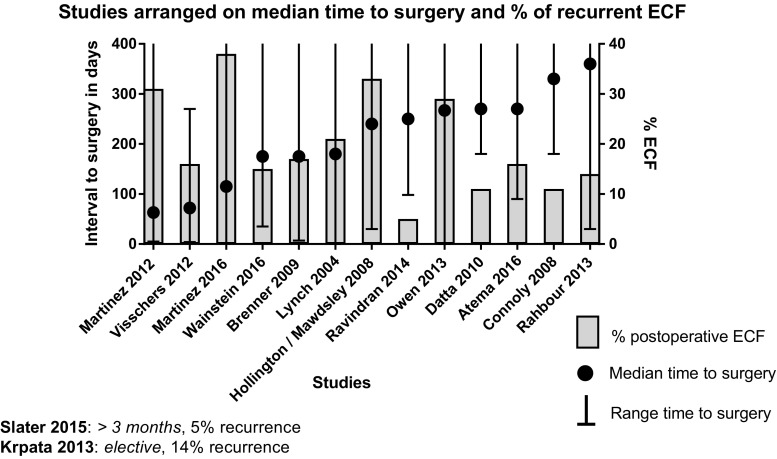
Median and range in time to surgery and ECF recurrence

Recurrence rates ranged from 5 to 38% (Table 1). In four studies, recurrence was defined as 30-day recurrence [25, 26, 28, 33], in two studies as 3-month recurrence [23, 27], and in one study as 6-month recurrence [32]. Eight other studies defined recurrence as recurrence at any point of time during follow-up (Table 1). The weighted pooled ECF recurrence rate was 19% (95% CI 15–24), *I* ^2^ 76% (Fig. 2). Figure 3 shows the median time to surgery and the minimum of the range (left y-axis) and the percentage of recurrent ECF (right y-axis). Lower recurrence rates were found in studies with a longer median time and/or, at the minimum of the range, a longer time interval to surgery. Lynch et al. [23] found an overall recurrence rate of 21%. A subgroup analysis of those patients who had undergone surgery within 3 months showed a recurrence rate of 28% (10 of 36) compared to a recurrence rate of 15% (7 of 114) in patients who had their operation after more than 3 months (*P* = .088). In a univariate analysis, Martinez [34] found that fistula surgery within 20 weeks was positively associated with mortality (*P* = 0.03). However, they did not find an association with fistula recurrence (*P* = 0.55). Brenner et al. [24] found that a waiting time of 36 weeks or longer was a significant risk factor for fistula recurrence. Patients who had undergone surgery after 36 weeks found a recurrence rate of 36%, compared to 12% in patients who had waited less than 36 weeks (*P* = .003). However, no statistical correction for confounding variables such as (co)morbidity was performed and these patients may have had significant morbidities delaying their surgery.

### Mortality

**Fig. 4:**
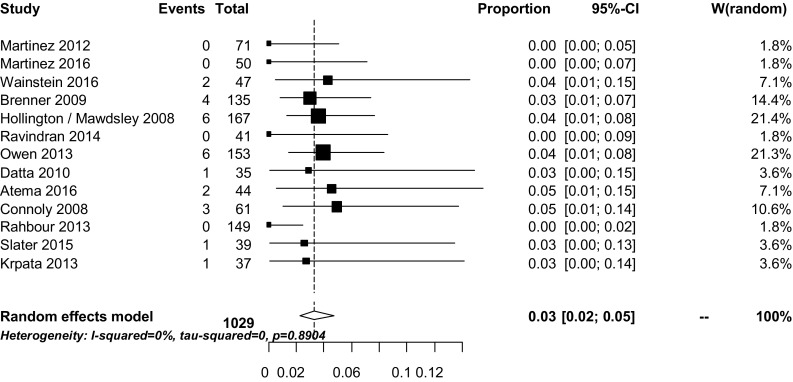
Weighted pooled short-term mortality rates

Short-term mortality rates were described in all studies except in two [23, 34]. Mortality rates ranged from 0 to 7% (Table 2). The overall weighted pooled mortality was 3% (95% CI 2–5), *I* ^2^ 0% (Fig. 4). The highest mortality rate (7%) was reported by the study with the shortest median waiting period to definitive surgery (median 72 days, range 4–270 days) among the included studies [16]. All other studies reported mortality rates of 5% or less. Lynch et al. [23] reported a 3-month mortality rate of 3%. Martinez [34] reported a mortality rate of 20%, but this was mortality at any point during follow-up and the total follow-up time was not recorded.

**Table 2:** Significant risk factors influencing recurrence and mortality extracted from included studies

ECF recurrence	Mortality
*Preoperative*	*Preoperative*
Complex fistula	Comorbidity
Inflammatory bowel disease	Low preoperative albumin
High-output fistula	Malnutrition
Preoperative diagnosis of short bowel syndrome	Fluid and electrolyte imbalance
Comorbidity	Transferred from other hospital
Interval between occurrence of fistula and operation > 36 weeks	TPN-induced cholestasis
> 1 year from diagnosis to OR	Preoperative CVL infection
Small bowel fistula	Gastric fistula
Preoperative serum C-reactive protein > 5 mg/dL	BMI < 20
	ASA 4
	Last abdominal procedure ≤ 20 weeks ago
	Uncontrollable sepsis
	Age ≥ 55 years
*Operative*	*Operative*
Wedge repair or oversewing	Wedge repair or oversewing
Stapled anastomosis	Operation > 8 h
Use of MESH	Estimate blood loss > 1L
Operation > 8 h	
Estimate blood loss > 325 mL	*Postoperative*
Fascia not closed	ECF recurrence after surgery
	Pneumonia
*Postoperative*	Unplanned intubation
Organ space SSI	Mechanical ventilation > 48 h
Mechanical ventilation > 48 h	Acute renal failure
Sepsis or shock	Sepsis or shock
Blood transfusion within 72 h	DVT
Length of stay > 30 days	Blood transfusion within 72 h

### Morbidity

There was a wide variation in the methods of reporting morbidity in the 15 studies. As listed in Table 1, morbidity was reported in only 10 of the 15 studies. Different classification systems were used, and therefore, pooling of morbidity data for meta-analysis was not possible. Six studies observed overall morbidity varying between 72 and 88% [16, 25, 27, 28, 30, 31]. One study reported a postoperative complication rate of 36% (scored according to the Clavien–Dindo classification Grade III or IV) [29]. Four studies reported on the occurrence of surgical site infections (SSIs) as described by the Center for Disease Control and Prevention [25, 28, 31, 33]. These ranged from 21% to 65%. Krpata et al. [34] found the highest percentage of SSIs, 65%; 19% of patients required an additional surgical intervention; and 19% required interventional radiology. Other studies reported less than 40% SSIs (range 21–38%). In one of these studies [31], 5% of the patients needed radiological drainage and in another study [32] 3% needed surgical re-interventions. More detailed information about the need for interventions for SSI was not provided. One study [23] did not report on morbidity but described a 6% reoperation rate after surgery.

### Hernia recurrence rate

**Fig. 5:**
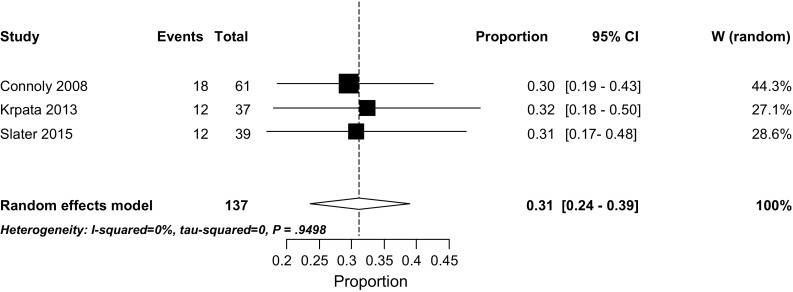
Weighted pooled hernia recurrence rates

Only three studies reported hernia recurrences rates [28, 31, 33]. The weighted pooled recurrence rate was 31% (95% CI 24.0–39.0) (Fig. 5). All studies involved large abdominal wall defects with simultaneous ECF takedown. Information on removal of infected mesh was not reported.

Krpata et al. [33] used a non-cross-linked biologic mesh in 97% of the cases. In 11%, a bridging mesh was required. They had a hernia recurrence rate of 32%, at a mean follow-up of 20 months. Connoly et al. [31] used either suture repair, suture repair with inlay prosthetic mesh, or prosthetic mesh alone and found an overall hernia recurrence rate of 29% (follow-up median 29 months). Slater et al. [28] found a hernia recurrence rate of 36% (mean follow-up of 63 months), using a component separation technique in 87% (34 of 39) of the patients, with a lightweight polypropylene mesh as reinforcement in 35% (12 of 34) of them. In that study, 10% of the patients had a bridging repair.

### Fistula closure rate

Twelve studies reported a total fistula closure rate that varied between 80 and 97% [16, 17, 24–27, 29, 30, 32–35]. Some of the patients needed up to three reoperations to achieve fistula closure; some patients died after recurrent fistulas; and others were left with a fistula because the risk of a reoperation was deemed too high.

### Enteral autonomy

Only four studies reported on patients regaining enteral autonomy. Datta [32] reported that of 12 patients receiving PN before definitive surgery, none remained dependent after surgery. In another study [27], in which 52% of the patients required preoperative PN, all but two could discontinue PN. In two other studies [25, 29], with 80 and 100% of the patients requiring PN preoperatively, respectively, 86 and 79% of the patients were able to discontinue PN postoperatively.

### Risk factors for recurrence and mortality

Many of the studies performed analyses to define risk factors for ECF recurrence, mortality, morbidity, hernia recurrence, or other factors negatively associated with healing. As ECF recurrence was the primary outcome of the present review, and mortality was also regarded as an important other outcome, risk factors that were found to be statistically significant for recurrence and mortality in one or more of the included articles have been summarized. Eight studies performed a specific analysis for risk factors for recurrence and mortality. For this review, these factors were divided into preoperative, operative, and postoperative risk factors and are summarized in Table 2.

Some of the risk factors are generally related to poor outcomes, such as postoperative complications or comorbidity. Other risk factors such as surgical technique are of more interest as they can be amended by the surgeon. Lynch et al. [25] found a fistula recurrence rate of 36% in those patients who underwent oversewing or wedge repair of an ECF, in contrast to a 17% recurrence in those patients who had undergone complete resection of the affected bowel segment. Brenner et al. [27] also found a fistula recurrence percentage of 22% for oversewing or wedge repair and 11% for complete segment resection. Although this effect on short-term fistula recurrence was not significant, it did have a significant effect on 1-year mortality (*P* = .003). Two studies showed that a stapled anastomosis was associated with a less favorable outcome [27, 28]. Brenner et al. [27] found a stapled anastomosis to be independently associated with ECF recurrence, and Owen et al. [28] found a significant negative effect of a stapled anastomosis during fistula surgery on 1-year mortality.

## Discussion

The management of ECF and/or EAF and the timing of IF surgery in patients with enteric fistula have changed over the past years. This systematic review and meta-analysis addresses the effect of postponed reconstructive surgery on outcome. The present review included fifteen studies; ten of which were retrospective cohort studies from single institutions. Recurrence rates varied considerably between studies, from 5 to 38% with a weighted pooled recurrence rate of 19% (95% CI 15–24). However, heterogeneity was considerable (*I* ^2^ 76%). Lower recurrence rates were found in studies with a longer median time and/or, at the minimum of the range, a longer time interval to surgery as shown in Fig. 3. The weighted pooled mortality rate was 4% and, although difficult to compare between studies, morbidity was considerable.

Present review aimed to define the optimal timing for enteric fistula surgery. Although the median time to surgery was reported in thirteen of the fifteen studies, a wide range of time to surgery within a study as well as a large variation in follow-up after surgery was present. For example, Rahbour et al. [29] described 149 patients with a median time to surgery of 360 days. The time to surgery, however, varied between 30 and 5100 days, and interquartile ranges were not provided. Therefore, we decided to plot the minimum of the range including the median in days to surgery for each study (Fig. 3) combined with ECF recurrence rates. Although a trend was found that a longer median time and/or, at the minimum of the range, a longer time interval to surgery has lower recurrence, it was not possible to define optimal timing of reconstructive surgery based on published data. Moreover, seven studies included patients with a period to surgery of less than 30 days [18, 25–29, 34]. This suggests that some of the patients in these studies may not have fulfilled the criteria of postponed surgery although mentioned in the present review’s methods section. Additionally, late referral to a specialized center after several surgical attempts might also have introduced a selection bias. Despite these difficulties, Fig. 3 indicates that a longer time to surgery is associated with lower recurrence. The study of Owen et al. [25] is the only outlier in the figure. However, as seen in the figure and in Table 1, this study also included patients with 0 days to surgery possibly explaining the higher recurrence rate.

The 30-day or in-hospital mortality rates were all 7% or less. This is much lower than reported in previous studies including both patients with acute and postponed surgery. These studies reported mortality rates between 10 and 20% after ECF surgery [36–39]. Different factors likely improved mortality rates, such as improved wound care, better intensive care facilities, and the possibility of radiological drainage. Postponed surgery will also have contributed to these improved results.

Stapled anastomosis was found a significant risk factor for fistula recurrence and 1-year mortality [28]. Based on the limited data in intestinal failure surgery and personal experience omitting bear staples in these types of abdomen, most of the specialists in the field believe that hand-sewn anastomoses are superior to stapled anastomosis in fistula surgery. No studies comparing the techniques for intestinal failure surgery have been published. However, most surgeons in the field feel that hand-sewn anastomoses are superior. Although the exact mechanism is unknown, it is believed that side (blind-end) staples can have large parts of bear staples which react with the environment and have a high risk of fistulation, certainly in contamination of microscopic leakage and/or intra-abdominal abscesses. Side-to-side length stapling leaves parts of the staples without initial peritoneal coverage. Finally, most of the patients undergo extensive adhesiolysis resulting in thickness of the bowel wall and, therefore, possibly stapled anastomoses are less safe.

Several limitations of this systematic review need to be addressed. The vast majority of studies were retrospective cohort studies of low to moderate quality. There was considerable clinical heterogeneity within and between cohorts due to different patient and fistula characteristics, surgical techniques, and follow-up making it difficult to compare studies. Importantly, only 6 of 15 included studies reported a follow-up of more than 3 months with respect to fistula recurrence; 4 studies reported only 30 day of fistula recurrence follow-up. Some of the studies did not mention follow-up time. This hampered a reliable estimate of fistula recurrence rates and in relation to timing of surgery, although most fistulas recur early in the postoperative period. As ECFs are relatively rare, most of the included studies came from specialized centers. Patients with less complex fistulas and in a better condition were less likely to have been referred. Therefore, the patients included in the present review reflect the more complicated end of the spectrum of enteric fistula patients. Despite these limitations, this review is the first to summarize the outcome of postponed ECF takedown as currently performed by most specialized centers.

Prospective and standardized data collection across IF centers is required before more precise recommendations can be made about optimal timing of reconstructive surgery for ECF and/or EAF. The optimal timing of IF surgery probably requires a time interval between 6 and 12 months after the last laparotomy because resolving abdominal infection, restoring nutritional state and homeostasis, and providing adequate wound care and muscle strength improving care like physiotherapy take a long period. This bridging-to-surgery approach was recently recommended and is now standard practice in IF centers [2].

## Appendix 1

See Table 3.

**Table 3:** Modified MINORS score

Item	Criterion	Option	Score
1	A clearly stated aim	Not reported	0
		Partially reported, no clear aim of study	1
		Clear aim of study	2
2	Inclusion of consecutive patients	Not reported	0
		> 25 patients, but unclear whether all were consecutive ECF patients	1
		> 25 patients and all were consecutive ECF patients	2
3	Prospective collection of data	Retrospective	0
		Prospective, not according to clearly stated protocol	1
		Prospective and according to protocol	2
4	Report of endpoints	Recurrence only	1
		Recurrence + one of secondary endpoints	2
5	Surgical technique reported	Not reported	0
		Incomplete	1
		Clear report of surgical technique	2
6	Time to elective surgery reported	Reported, but wide range with cases < 3 months	0
		Only elective mentioned	1
		Reported, small range	2
7	Follow-up time appropriate	Not reported	0
		Mean/median ≤ 6 months	1
		Mean/median > 6 months	2

## Appendix 2

### Search August 24, 2016

#### PubMed (1223)

(“Intestinal Fistula”[Mesh:noexp] OR enterocutaneous fistula*[tiab] OR enteric fistula*[tiab] OR entero-cutaneous fistula*[tiab] OR enteroatmospheric fistula*[tiab] OR entero-atmospheric fistula*[tiab] OR small bowel fistula*[tiab] OR colonic fistula*[tiab] OR intestinal failure*[tiab] OR abdominal wall defect*[ti] OR open abdomen[ti]) AND (surgical treatment[tiab] OR repair[tiab] OR closure[tiab] OR surgical management[tiab] OR clinical management [tiab] OR abdominal surgery[tiab]) AND (“Postoperative Complications”[Mesh] OR “Recurrence”[Mesh] OR “Mortality”[Mesh] OR mortality[tiab] OR morbidity[tiab] OR recurrence[tiab] OR complication*[tiab]) NOT (“Case Reports” [Publication Type] OR “Letter” [Publication Type]).

#### Embase (711)

((intestine fistula/or (enterocutaneous fistula* or enteric fistula* or entero-cutaneous fistula* or enteroatmospheric fistula* or entero-atmospheric fistula* or intestinal failure* or small bowel fistula* or colonic fistula*).ti,ab,kw. or abdominal wall defect*.ti. or open abdomen.ti.) and (Surgical treatment or Surgical management or clinical management or abdominal surgery or (ECF adj3 closure) or (fistula* adj3 closure*) or (enterocutaneous fistula* adj3 closure*) or (ECF adj3 surgery) or (enterocutaneous fistula* adj3 surger*) or (ECF adj3 treatment*) or (enterocutaneous fistula* adj3 treatment*)).ti,ab,kw. and (exp postoperative complication/or exp recurrent disease/or exp mortality/or morbidity/or (mortalit* or morbidit* or recurrence or complication*).ti,ab,kw.)) not (case report/or letter/).

#### Cochrane (49)

(enterocutaneous fistula* or enteric fistula* or entero-cutaneous fistula* or enteroatmospheric fistula* or entero-atmospheric fistula* or intestinal failure* or small bowel fistula* or colonic fistula* or abdominal wall defect* or open abdomen) AND (Surgical treatment or Surgical management or clinical management or abdominal surgery) AND (mortalit* or morbidit* or recurrence or complication*).
